# Development and Validation of an Early Postoperative Risk Stratification Model for Hydrocephalus Following Aneurysmal Subarachnoid Hemorrhage

**DOI:** 10.1002/brb3.71869

**Published:** 2026-10-28

**Authors:** Fuguang Li, Kunxu Fu, Shifang Zhou, Zipeng Gong, Chaoyang Du, Yuxi Zhao, Weilong Yang, Chunyu Zhang, Wenke Zhou, Wenchao Li

**Affiliations:** ^1^ Department of Neurosurgery The First Affiliated Hospital of Henan Medical University; Henan medical Key Laboratory of Neurology, Henan Joint International Laboratory of Neurorestoratology for Senile Dementia, Henan Engineering Technology Research Center of Neurorestoratology ,Henan Engineering Laboratory of Neurorestoratology,Henan Key Laboratory of Neurorestoratology and Protein modification, First Affiliated Hospital of Henan Medical University, Xinxiang, Henan xinxiang city China

**Keywords:** Aneurysmal subarachnoid hemorrhage, hydrocephalus, machine learning, Shapley additive explanations interpreter

## Abstract

**Background:**

Hydrocephalus is a common complication after aneurysmal subarachnoid hemorrhage (aSAH). This study aimed to identify perioperative risk factors and develop an early postoperative risk stratification model to support postoperative monitoring rather than purely preoperative prediction.

**Methods:**

Employing a retrospective case‐control design, clinical data from 331 patients with aSAH treated at the First Affiliated Hospital of Henan Medical University between August 1, 2018, and December 31, 2024, were collected and analyzed. The primary endpoint was postoperative hydrocephalus occurring within 3 weeks after aneurysm treatment, diagnosed according to combined radiological and clinical criteria. The least absolute shrinkage and selection operator regression method was employed to screen risk factors. Multiple machine‐learning models were compared, and Shapley additive explanations (SHAP) analysis was used for individualized model interpretation.

**Results:**

The admission Glasgow Coma Scale score, intraoperative external ventricular drainage/lateral ventricular drainage, and intraventricular hemorrhage were identified as key variables associated with postoperative hydrocephalus. The logistic regression model emerged as the optimal model; locked test set area under the curve (95% CI): 0.891 (0.739–1.000); accuracy: 0.720 (0.688–0.775); sensitivity: 0.899 (0.823–0.987); specificity: 0.683 (0.635–0.740).

**Conclusion:**

The logistic regression model with SHAP‐based interpretation may support early postoperative risk stratification for hydrocephalus after aSAH. Given its modest positive predictive value, the model should be used to triage patients for intensified monitoring and timely reassessment.

## Graphical abstract

This study developed and validated a machine learning‐based prediction model for postoperative hydrocephalus following aneurysmal subarachnoid hemorrhage (aSAH). Using retrospective data from 331 patients, we identified four key predictors: admission GCS score, admission Hunt‐Hess grade, intraoperative external ventricular drainage (EVD), and concomitant intraventricular hemorrhage (IVH). A logistic regression model demonstrated superior predictive performance (AUC = 0.883) and was interpreted using SHAP to provide individualized risk assessments. The model facilitates early identification and intervention for high‐risk aSAH patients, supporting personalized clinical decision‐making

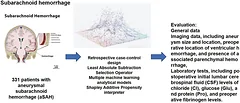

## 1 Introduction

Subarachnoid hemorrhage (SAH) is an acute cerebrovascular disorder caused by the rupture of blood vessels on the brain's surface. Blood directly enters the subarachnoid space, resulting in severe neurological impairment. Epidemiological studies indicate that the global annual incidence of SAH is approximately 6–9 cases per 100,000 people, accounting for approximately 5% of all stroke cases. Among these, aneurysmal SAH (aSAH), caused by ruptured aneurysms, constitutes approximately 85% of all SAH cases (Sweeney et al. 2016). Hydrocephalus is a common complication of aSAH, often manifesting postoperatively as increased intracranial pressure, pupillary abnormalities, worsening consciousness impairment, and, in the most severe cases, life‐threatening consequences. Early identification of risk factors and development of predictive models are crucial for enhancing early prevention of postoperative hydrocephalus in patients with aSAH. Although many studies employ traditional predictive models, the application of machine learning (ML) remains underutilized. Therefore, this study utilizes multiple ML classification models to construct predictive frameworks (Culebras et al. 2025; Adams and Leveson 2012).

By collecting and organizing clinical data from patients who developed postoperative hydrocephalus following aSAH, we analyzed risk factors for postoperative hydrocephalus. This enables early intervention and focused monitoring for patients at risk of developing hydrocephalus in the near term. The advantage of ML lies in its status as an emerging medical field, representing a powerful set of algorithms capable of representing, adapting to, learning from, predicting, and analyzing data. Furthermore, ML is considered the future of personalized medicine and computer‐aided diagnosis in biomedical research (Greener et al. 2022; Bazoukis et al. 2022). The application significance of ML models lies in demonstrating accurate personalized predictions and promising clinical utility (Padula et al. 2024). However, evidence of its application in real clinical settings and interpretable risk prediction models remains limited (Vayena et al. 2018; Shortliffe and Sepúlveda 2018).

Therefore, we incorporate the Shapley additive explanations (SHAP) interpretation tool to provide intuitive explanations for patient risk predictions (Lundberg et al. 2020). This study employs traditional regression and ML methods to develop and validate an early postoperative risk stratification model for hydrocephalus following aSAH. Based on data from 331 patients, the final logistic regression model identifies the admission Glasgow Coma Scale (GCS) score, intraoperative external ventricular drainage (EVD), and intraventricular hemorrhage (IVH) as key variables associated with postoperative hydrocephalus. Importantly, because the candidate variables included admission, intraoperative, and early postoperative information, this model should not be interpreted as a purely preoperative prediction model. Instead, it is intended to support dynamic risk assessment after aneurysm treatment and to help clinicians identify patients who may benefit from intensified monitoring, repeated neuroimaging, or timely evaluation for cerebrospinal fluid (CSF) diversion.

## 2 Materials and Methods

### 2.1 Materials

#### 2.1.1 Study Population

A total of 331 patients with aSAH admitted to the First Department of Neurosurgery at the First Affiliated Hospital of Henan Medical University between August 1, 2018, and December 31, 2024, were enrolled. This study was conducted in accordance with the Declaration of Helsinki and approved by the Ethics Committee of the First Affiliated Hospital of Henan Medical University (Approval Number: EC‐025‐10). Because this was a retrospective study using de‐identified clinical data, the requirement for written informed consent was waived by the ethics committee.

#### 2.1.2 Inclusion and Exclusion Criteria

Inclusion criteria: (1) intracranial aneurysm confirmed by computed tomography (CT) angiography, with imaging evidence of aSAH; (2) complete demographic, clinical, and imaging records. Exclusion criteria: (1) incomplete medical records; (2) poor surgical candidacy due to severe comorbidities or conditions, such as pacemakers, cranial defects, or epilepsy; (3) indeterminate postoperative hydrocephalus status; and (4) postoperative complications, such as intracranial hemorrhage, major cerebral infarction, or infection.

### 2.2 Methods

#### 2.2.1 Patient Grouping and Diagnosis for Aneurysmal Subarachnoid Hemorrhage

The primary endpoint was postoperative hydrocephalus occurring within 3 weeks after aneurysm treatment. Postoperative hydrocephalus was diagnosed when both radiological evidence of ventricular enlargement and compatible clinical deterioration were present. Radiological hydrocephalus was defined as ventricular enlargement on follow‐up CT compared with the preoperative or immediate postoperative CT, including a bicaudate index exceeding the age‐adjusted upper limit or a relative bicaudate index > 1.0, enlargement of the temporal horns or progressive enlargement of the lateral and/or third ventricles requiring clinical concern for impaired CSF circulation. Clinical criteria included newly developed or aggravated symptoms suggestive of hydrocephalus, such as decreased level of consciousness, headache, nausea or vomiting, gait instability, urinary incontinence, or cognitive decline. These symptoms were considered attributable to hydrocephalus only when they could not be better explained by rebleeding, major cerebral infarction, infection, electrolyte disturbance, or other postoperative complications. Therefore, the diagnosis required at least one radiological criterion together with at least one compatible clinical criterion. Final adjudication was performed by two experienced neurosurgeons, based on serial CT findings and clinical records, with any disagreements resolved through discussion with a senior neurosurgeon (Dupont and Rabinstein 2013).

Cranial CT was routinely performed before treatment, within 24 h after aneurysm treatment, and subsequently according to clinical condition during hospitalization. Additional CT scans were performed when neurological deterioration or symptoms suggestive of hydrocephalus occurred. The diagnosis of postoperative hydrocephalus was adjudicated by two experienced neurosurgeons based on imaging findings and clinical records. Disagreements were resolved by discussion with a senior neurosurgeon. Because of the retrospective nature of the study, outcome assessors were not formally blinded to all clinical information; however, the endpoint was determined before model construction to reduce assessment bias.

Patients were divided into the hydrocephalus group and the non‐hydrocephalus group according to whether they met the above endpoint within 3 weeks after aneurysm treatment (de Hond et al. 2022; Panciani et al. 2025; Eide et al. 2020).

#### 2.2.2 Data Collection

This study included 21 indicators: (1) general data, including age, gender, history of hypertension, smoking history, history of diabetes, GCS score at admission, Hunt–Hess classification at admission, modified Fisher classification at admission, surgical approach, whether lateral ventriculostomy and craniostomy were performed intraoperatively, postoperative lumbar puncture, and postoperative lumbar cisternal drainage; (2) imaging data, including aneurysm size and location, preoperative location of ventricular hemorrhage, and presence of associated parenchymal hemorrhage; (3) laboratory tests, including postoperative initial lumbar CSF levels of chloride (Cl), glucose (Glu), and protein (Pro), and preoperative fibrinogen levels. Because the candidate variables included admission, intraoperative, and early postoperative information, the present model was defined as an early postoperative risk stratification model rather than a purely preoperative prediction model. Its intended clinical use was to identify patients requiring closer postoperative surveillance, repeated imaging, or timely evaluation for CSF diversion after aneurysm treatment.

### 2.3 Construction and Evaluation of the Predictive Model

#### 2.3.1 Internal Validation and Test‐Set Evaluation

The analysis pipeline was clarified as follows. First, the entire cohort of 331 patients was randomly divided once into a training set and a locked test set at an approximate ratio of 7:3. The locked test set included 100 patients and was not used for variable selection, cutoff determination, model training, or hyperparameter selection. All preprocessing steps, including receiver operating characteristic (ROC)‐derived cutoff determination, least absolute shrinkage and selection operator (LASSO)‐based variable selection and model fitting, were performed within the training set. Internal validation was performed using 10‐fold cross‐validation within the training set. After the final model was determined, its performance was evaluated once in the locked test set. Therefore, the cross‐validation results were used for internal validation, whereas the locked test‐set results were used for reporting final model performance.

#### 2.3.2 Model Timing and Intended Use

The intended prediction time point of this model was the immediate postoperative period after aneurysm treatment, when admission, imaging, intraoperative, and early postoperative information had become available. Therefore, this model was not designed as a purely preoperative prediction model. Instead, it was developed as an early postoperative risk stratification model to identify patients who may require closer monitoring for hydrocephalus within 3 weeks after treatment.

#### 2.3.3 Binarization of Continuous Variables

The threshold determination followed this rigorous statistical process.

Least absolute shrinkage and selection operator regression: This was performed using a binomial model. The minimum mean squared error lambda was 0.021, and the lambda selected by the one‐standard‐error rule was 0.072. To obtain a more parsimonious and clinically interpretable model, the one‐standard‐error lambda was used for final variable selection. The final selected variables were admission GCS score, admission Hunt–Hess grade, lateral ventricular drainage/EVD, and IVH. Given the limited number of outcome events, this LASSO‐based selection process was interpreted as exploratory model reduction rather than causal inference. Detailed parameters can be found in the supplementary materials (Table S1).

Threshold calculation: Based on data from the training set (*n* = 231), ROC curves were plotted for each continuous variable predicting postoperative hydrocephalus. The optimal cutoff value (threshold) was defined as the value that maximized the Youden Index (*J*), which is calculated as *J* = Sensitivity + Specificity − 1.

Threshold stability assessment: To evaluate the stability of the estimated thresholds, internal validation was performed using the bootstrap resampling method. A total of 1000 bootstrap samples with replacement were drawn from the training set. For each sample, the calculations from Step 1 were repeated to obtain a threshold estimate. Finally, the optimal threshold based on the original training set was reported, along with the 95% CI (calculated using the percentile method) derived from the threshold distribution obtained through 1000 bootstrap samples. All analyses were performed with a random seed of 42 to ensure reproducibility.

The final model equation: logit(*P*) = −0.801 − 0.496 × GCS + 0.003 × Hunt–Hess − 0.674 × EVD + 0.885 × IVH, where *P* represents the predicted probability of postoperative hydrocephalus. The decision threshold used for binary classification in the locked test set was 0.198. At this threshold, the locked test‐set area under the ROC curve (AUC) was 0.891, accuracy was 0.720, sensitivity was 0.889, and specificity was 0.683.

Final assignment: The details are available in the supporting materials (Sauerbrei et al. 2023; Obuchowski and Bullen 2018; Muschelli 2020; Belkin et al. 2019; Claassen and Park 2022). The authors acknowledge that dichotomizing continuous variables may lead to loss of information and reduced statistical efficiency. In this study, dichotomization was performed primarily to improve clinical interpretability and facilitate bedside risk stratification. The selected cutoffs were derived from the training set using ROC analysis and internally assessed by bootstrap resampling.

Sensitivity analysis for dichotomized GCS score: Because dichotomization of continuous variables may result in information loss, a sensitivity analysis was performed to evaluate the robustness of the primary model. In this analysis, the final logistic regression model was refitted by treating admission GCS score as a continuous variable rather than using the ROC‐derived cutoff of 11. The remaining variables, including Hunt–Hess grade, intraoperative EVD/lateral ventricular drainage, and IVH, were kept consistent with the primary model. Model performance was compared with that of the primary dichotomized model using AUC, accuracy, sensitivity, specificity, positive predictive value, negative predictive value, F1 score, Brier score, calibration slope, and calibration intercept. Given the limited number of outcome events, restricted cubic spline modelling was not performed to avoid increasing model complexity and overfitting risk.

### 2.4 Statistical Analysis

All statistical analyses were performed using R version 4.2.3 and Python version 3.11.4. The LASSO regression was performed using R version 4.3.0 with the glmnet package version 4.1.8. A random seed of 42 was used to improve reproducibility. First, missing data were assessed before model development. Only three variables had missing values: postoperative CSF Cl, Glu, and Pro levels, each with 44 missing values, corresponding to a missing rate of 13.3%. Because the missingness was considered clinically plausible and no systematic pattern was identified, the data were assumed to be missing at random (MAR). The K‐nearest neighbor (KNN) single imputation method was used to impute these missing values. To avoid information leakage, the imputation model was fitted only within the training set, and the learned imputation procedure was then applied to the locked test set. Outcome status was not used as a predictor in the imputation process. Complete‐case sensitivity analysis was considered but was limited by the reduced sample size; therefore, the imputed dataset was used for the primary analysis. The use of KNN imputation was chosen because it can preserve local similarity among clinical and laboratory variables without imposing a parametric distributional assumption. Given the low missing rate and the pattern of MAR, single imputation was employed to fill these gaps. The details can be seen in the supporting materials. This study was reported in accordance with the Transparent Reporting of a Multivariable Prediction Model for Individual Prognosis or Diagnosis plus Artificial Intelligence (TRIPOD+AI) statement for clinical prediction model studies using regression or ML methods. A completed TRIPOD+AI checklist is provided as Supplementary Table S2.

## 3 Results

### 3.1 Preliminary Screening of Potential Risk Factors Using Univariate Analysis

A total of 331 patients were included, of whom 66 (19.9%) developed postoperative hydrocephalus. The cohort was randomly split into a training set (*n* = 231, 19.5% hydrocephalus) and a locked test set (*n* = 100, 20.0% hydrocephalus), with balanced outcome distribution. Univariate analysis identified several factors significantly associated (*p* < 0.05) with hydrocephalus, including admission Hunt–Hess and Fisher grades, aneurysm size, surgical approach, intraoperative EVD, IVH, postoperative lumbar puncture, intraoperative endoscopic fistulization, admission GCS score, and postoperative CSF Glu and Pro levels (Table 1). Variables with *p* < 0.15, including IVH, were retained for further analysis.

**TABLE 1:** Results of single‐factor analysis of independent variables.

Variable	Categorical variable	No Hydrocephalus Group (*n* = 265)	Hydrocephalus Group (*n* = 66)	*t/x* ^2^	p
Gender	Female	175 (66.038)	42 (63.636)	0.135	0.713
	Male	90 (33.962)	24(36.364)		
Age		59.000 [51.000, 68.000]	63.000 [50.000, 69.000]	− 0.572	0.568
Glasgow Coma Scale score at admission (*n*, %)		43 (16.226)	45 (68.182)	82.735	<0.001
History of hypertension	No	112 (42.264)	26 (39.394)	0.179	0.672
	Yes	153 (57.736)	40 (60.606)		
Diabetes history	No	252 (95.094)	60 (90.909)	1.711	0.191
	Yes	13 (4.906)	6 (9.091)		
Smoking history	No	217 (81.887)	51 (77.273)	0.730	0.393
	Yes	48 (18.113)	15 (22.727)		
Hunt‐Hess classification at admission	Level 1	65 (24.528)	1 (1.515)	71.301	<0.001
	Level 2	86 (32.453)	6 (9.091)		
	Level 3	34 (12.830)	4 (6.061)		
	Level 4	64 (24.151)	34 (51.515)		
	Level 5	16 (6.038)	21 (31.818)		
Fisher classification at admission	Grade 1	68 (25.660)	1 (1.515)	78.332	<0.001
	Level 2	62 (23.396)	1 (1.515)		
	Level 3	56 (21.132)	5 (7.576)		
	Level 4	79 (29.811)	59 (89.394)		
Aneurysm size	≤10mm	161 (60.755)	31 (46.970)	4.122	0.042
	>10mm	104 (39.245)	35 (53.030)		
Aneurysm location	Anterior circulation	246 (92.830)	60 (90.909)	0.279	0.597
	Post‐cycle	19 (7.170)	6 (9.091)		
Surgical approach	Surgical clamping	118 (44.528)	41 (62.121)	6.552	0.010
	Vascular Intervention	147 (55.472)	25 (37.879)		
Intraoperative endoscopic fistulization	No	226 (85.283)	45 (68.182)	10.412	0.001
	Yes	39 (14.717)	21 (31.818)		
Intraoperative EVD	No	27 (10.189)	37 (56.061)	71.289	< 0.001
	Yes	238 (89.811)	29(43.939)		
Combined IVH	No	190 (71.698)	8 (12.121)	78.021	< 0.001
	Yes	75 (28.302)	58(89.879)		
Combined ICH	No	236(89.057)	54(81.818)	2.551	0.110
	Yes	29 (10.943)	12 (18.182)		
Postoperative lumbar puncture	No	41 (15.472)	3 (4.545)	5.473	0.019
	Yes	224 (84.528)	63 (95.455)		
Postoperative lumbar cistern drainage	No	257 (96.981)	58 (87.879)	9.517	0.002
	Yes	8 (3.019)	8 (12.121)		
Postoperative CSF CI concentration mmol/L		124.600 [119.900, 128.000]	123.900 [118.400, 127.500]	0.864	0.388
Postoperative CSF GLU concentration (mmol/L)		3.100 [2.360, 3.680]	2.780 [1.880, 3.610]	2.222	0.026
Postoperative CSF protein (Pro) level (mg/L)		820.000 [501.900, 1600.340]	1695.470 [747.800, 3654.200]	−3.918	< 0.001
Admission fibrinogen		290.500 [246.700, 332.550]	284.600 [239.400, 343.200]	−0.244	0.808

*Note*: GCS score on admission: Glasgow Coma Scale score on admission; Admission Hunt‐Hess score: No Chinese equivalent; Admission Fisher score: No Chinese equivalent; Intraoperative EVD: Intraoperative external ventricular drainage; Concurrent IVH: Concurrent intraventricular hemorrhage; Concurrent ICH: Concurrent intracerebral hemorrhage; Postoperative CSF levels of CL, GLU, and Pro: chloride, glucose, and protein concentrations, respectively.

### 3.2 Comparison of Baseline Data Between Training and Locked Test Sets

This study included 331 patients, divided into training and locked test sets at a 1:0.3 ratio. The selected independent variables were incorporated into the analysis. The results indicated the following: Hunt–Hess classification at admission, Fisher classification at admission, aneurysm size, surgical approach, presence of endplate fenestration during surgery, presence of EVD during surgery, presence of IVH, presence of ICH, postoperative lumbar puncture, postoperative lumbar cisternal drainage, admission GCS score, Glu level in the first postoperative lumbar CSF sample, and Pro level in the first postoperative lumbar CSF sample, and presence of parenchymal hemorrhage and presence of lateral ventriculostomy showed no significant difference (Table S3).

### 3.3 Binarization of Continuous Variables and Threshold Determination

Receiver operating characteristic analysis was used to classify admission GCS scores, postoperative CSF Glu levels, and postoperative CSF Pro levels, with postoperative hydrocephalus occurrence as the outcome. Based on the maximum Youden index, the cutoff values were determined as follows: admission GCS score, 11 points; postoperative CSF Glu level, 2.00 mmol/L; and postoperative CSF Pro level, 1226.99 mg/L (Table S4, Figure 1). An admission GCS > 11 was assigned a value of 1, and an admission GCS ≤ 11 was assigned a value of 0; CSF Glu content > 2.00 mmol/L was assigned a value of 1, and CSF Glu content ≤ 2.00 mmol/L was assigned a value of 0; CSF Pro content ≤ 1226.99 mg/L was assigned a value of 1, and CSF Pro content > 1,226.99 mg/L was assigned a value of 0 (Table 2).

**FIGURE 1:**
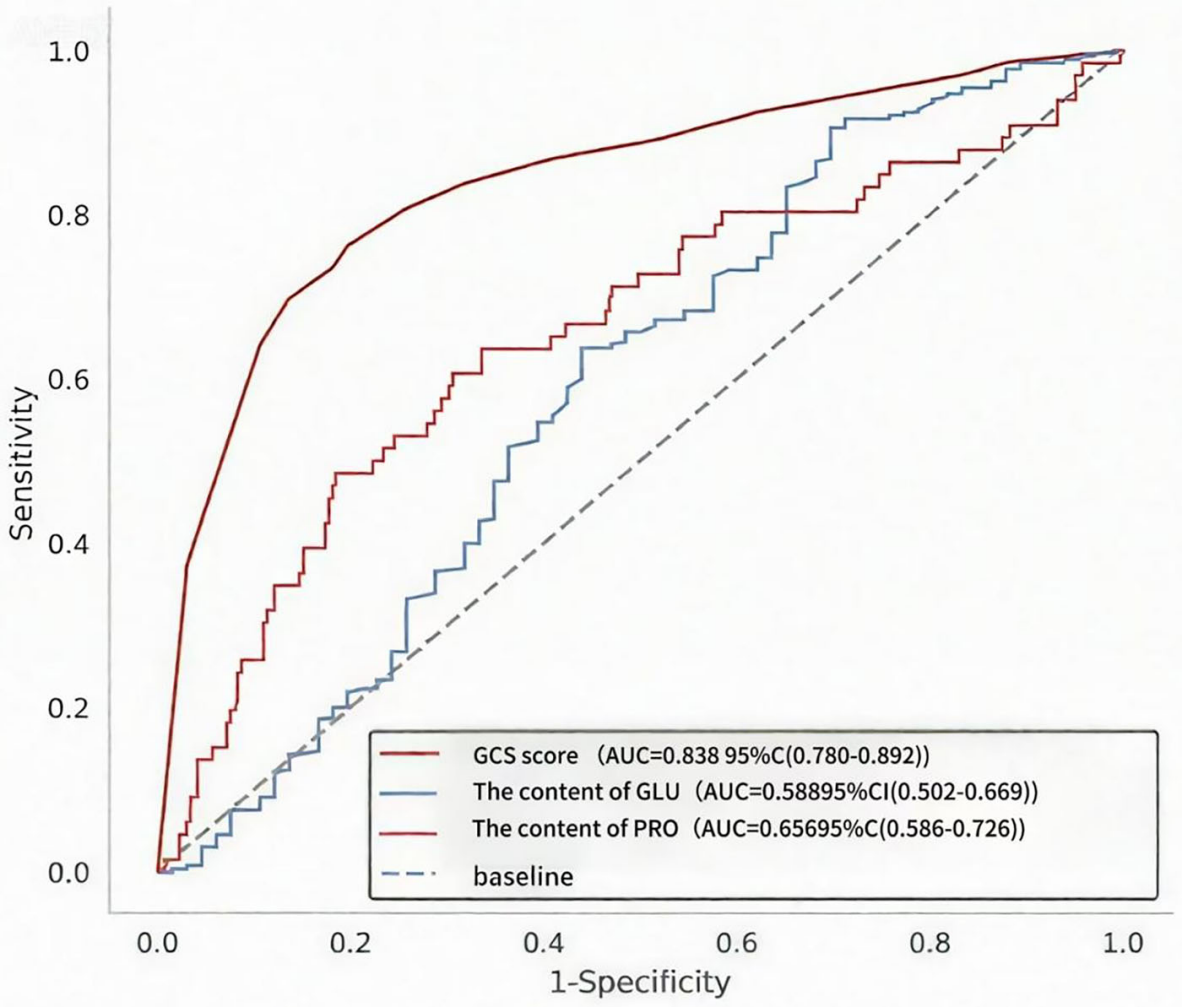
Receiver operating characteristic curves for the Glasgow Coma Scale score, postoperative cerebrospinal fluid (CSF) glucose concentration and postoperative CSF protein level.

**TABLE 2:** Assignment Table.

Variable name	Assignment
Admission GCS Score	
≤11 points	0
>11 points	1
Postoperative cerebrospinal fluid GLU concentration	
≤2.000 mmol/L	0
>2.000 mmol/L	1
Postoperative cerebrospinal fluid protein concentration	
≤1226.990 mg/L	0
>1226.990 mg/L	1

### 3.4 Using Least Absolute Shrinkage and Selection Operator Regression to Screen Risk Factors

A LASSO regression analysis was performed on the remaining independent variables, with postoperative hydrocephalus occurrence as the dependent variable. Since LASSO regression automatically handles multicollinearity among independent variables through L1 regularization and shrinks the coefficients of partially collinear variables to zero, traditional variance inflation factor diagnostics were not performed; instead, LASSO's built‐in feature selection mechanism was utilized (Eide et al. 2020). Results showed (lambda = 0.064, minimum mean squared error) that 14 independent variables were reduced to 4, namely, admission GCS score, admission Hunt–Hess grade, intraoperative EVD placement, and presence of IVH (Table S5, Figure 2).

**FIGURE 2:**
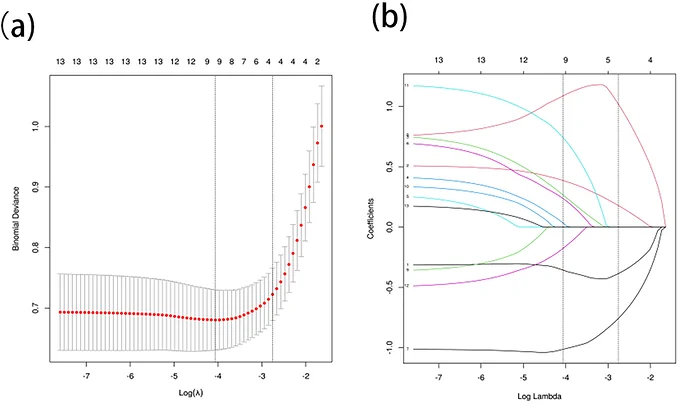
Feature selection using least absolute shrinkage and selection operator regression analysis. (a) Penalty profile plot of variable coefficients for four potential influencing factors. (b) Binomial bias plot for optimal penalty coefficient lambda.

### 3.5 Comprehensive Comparison of Multi‐Model Classification

Eight prediction models were retrained 10 times, with AUC as the evaluation metric. On the training set, XGBoost, Random Forest, and LightGBM achieved the highest AUC values; on the locked test set, logistic regression and Gaussian Naive Bayes performed best (Figures 3a,b). As AUC alone does not fully reflect clinical utility, model performance was further evaluated using decision curve analysis (DCA), calibration curves and precision–recall (PR) curves. The DCA demonstrated that across a threshold probability range of 5%–50%, the logistic regression, XGBoost, and LightGBM models consistently provided higher net benefit than the “treat‐all” or “treat‐none” strategies (Figure 3c). Among them, the logistic regression model achieved the highest net benefit at thresholds of 10%, 20%, and 30%, reaching a maximum of 0.132 at 20% (Table 3). This corresponds to identifying 13.2 additional true positive cases per 100 patients without increasing unnecessary interventions. However, the clinical utility suggested by DCA should be interpreted together with the modest positive predictive value. At the selected decision threshold of 0.198, the positive predictive value was 0.381, indicating that approximately 62% of patients who were model positive may not develop postoperative hydrocephalus. Therefore, a high‐risk model output should not be regarded as a diagnosis or as an indication for automatic intervention. Instead, it should prompt intensified postoperative surveillance, repeated neurological assessment, timely cranial CT re‐evaluation, and clinical review for possible CSF diversion when appropriate. Calibration curves further confirmed that the logistic regression model exhibited minimal prediction error (Figure 3d), indicating excellent probability calibration.

**FIGURE 3:**
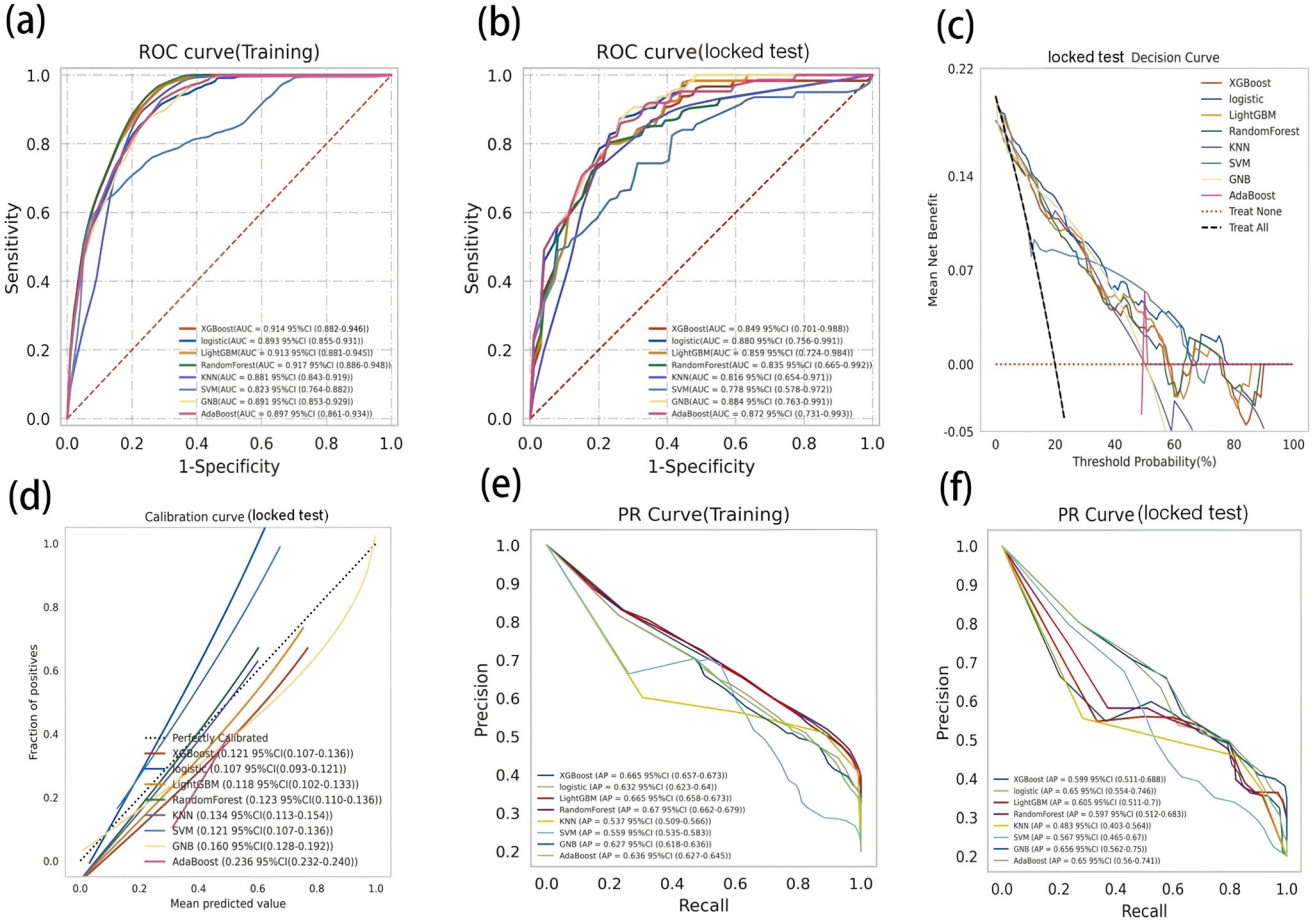
Comprehensive multi‐model performance analysis. (a) Training set receiver operating characteristic (ROC) and area under the curve (AUC). (b) Locked test set ROC and AUC. Patients with aneurysmal subarachnoid hemorrhage were split once into a training set and a locked test set at an approximate ratio of 7:3; 10‐fold cross‐validation was performed within the training set. (c) Decision curve analysis for the locked test set. X‐axis: decision threshold probability (Pt, range 0%–50%); y‐axis: net benefit. The grey solid line represents the ‘all intervention’ strategy; the thick black solid line represents the ‘all no intervention’ strategy; colored solid lines represent net benefit curves for different models. Within the Pt range of approximately 5%–50%, the logistic regression, XGBoost and LightGBM models demonstrate clinical utility. Specific values are shown in Table 3. (d) Locked test set calibration curves. Solid lines represent Loess‐fitted curves for each model; closer proximity to the dotted diagonal indicates better calibration. Quantitative calibration metrics (Brier score, calibration slope, calibration intercept) for each model are listed in Table S5. (e) Training set precision–recall (PR) curves and average precision (AP). (f) Locked test set PR curve and AP. The vertical axis represents precision, and the horizontal axis represents recall. If one model's PR curve fully encompasses another model's PR curve, the former is considered superior. A higher AP value indicates better model performance. Each model is represented by a distinct color in the figure and presented as a mean with a 95% CI.

**TABLE 3:** Net benefit of each model at specific decision thresholds on the Locked test set.

Model	Net Benefit @ Pt=10%	Net Benefit @ Pt=20%	Net Profit @ Pt=30%
Logistic	0.185	0.132	0.080
XGBoost	0.178	0.125	0.072
LightGBM	0.181	0.128	0.075
Random Forest	0.162	0.110	0.058
AdaBoost	0.170	0.118	0.065
SVM	0.168	0.115	0.063
KNN	0.165	0.112	0.060
GNB	0.155	0.102	0.050
All Intervention Strategies	0.100	0.000	−0.100
All non‐intervention strategies	0.000	0.000	0.000

As shown in Table S6, the logistic regression model demonstrated optimal calibration performance on the locked test set, exhibiting the lowest Brier score and calibration slope and intercept closest to ideal values, consistent with the intuitive display of the calibration curve. The PR curve indicates that logistic regression achieved the highest average precision on the locked test set (Figures 3e, f). Following comprehensive evaluation of discrimination, calibration, decision‐curve analysis, PR performance, and interpretability, the logistic regression model showed the most stable overall performance and was selected as the final model.

### 3.6 Construction and Validation of the Optimal Model

The final LASSO‐selected logistic model included four variables: admission GCS score, admission Hunt–Hess grade, lateral ventricular drainage/EVD, and IVH. Under the one‐standard‐error lambda, the model intercept was −0.801. The coefficients were −0.496 for admission GCS score, 0.003 for Hunt–Hess grade, −0.674 for lateral ventricular drainage/EVD, and 0.885 for IVH. The final logistic regression classifier was further evaluated by 10‐fold cross‐validation and an independent test set. After variable selection and model training on the training set, the final logistic regression model was internally validated with 10‐fold cross‐validation and then evaluated on the held‐out locked test set. In the internal cross‐validation, the mean AUC was 0.893. In the locked test set, the final model achieved an AUC of 0.891, accuracy of 0.720, sensitivity of 0.889, specificity of 0.683, positive predictive value of 0.381, negative predictive value of 0.966, F1 score of 0.533, and kappa of 0.376. These locked test‐set results were used as the primary estimate of final model performance (Figures 4a–c). The AUC values for the training locked test sets all stabilized around 0.9, indicating the model's high predictive accuracy. When the locked test‐set AUC is less than the test‐set AUC or the difference between them is <10%, it indicates a good model fit. As shown by the learning curve in Figure 4d, the training and locked test‐set exhibit high fit and good stability (Muschelli 2020). Thus, the logistic regression model is suitable for this dataset.

**FIGURE 4:**
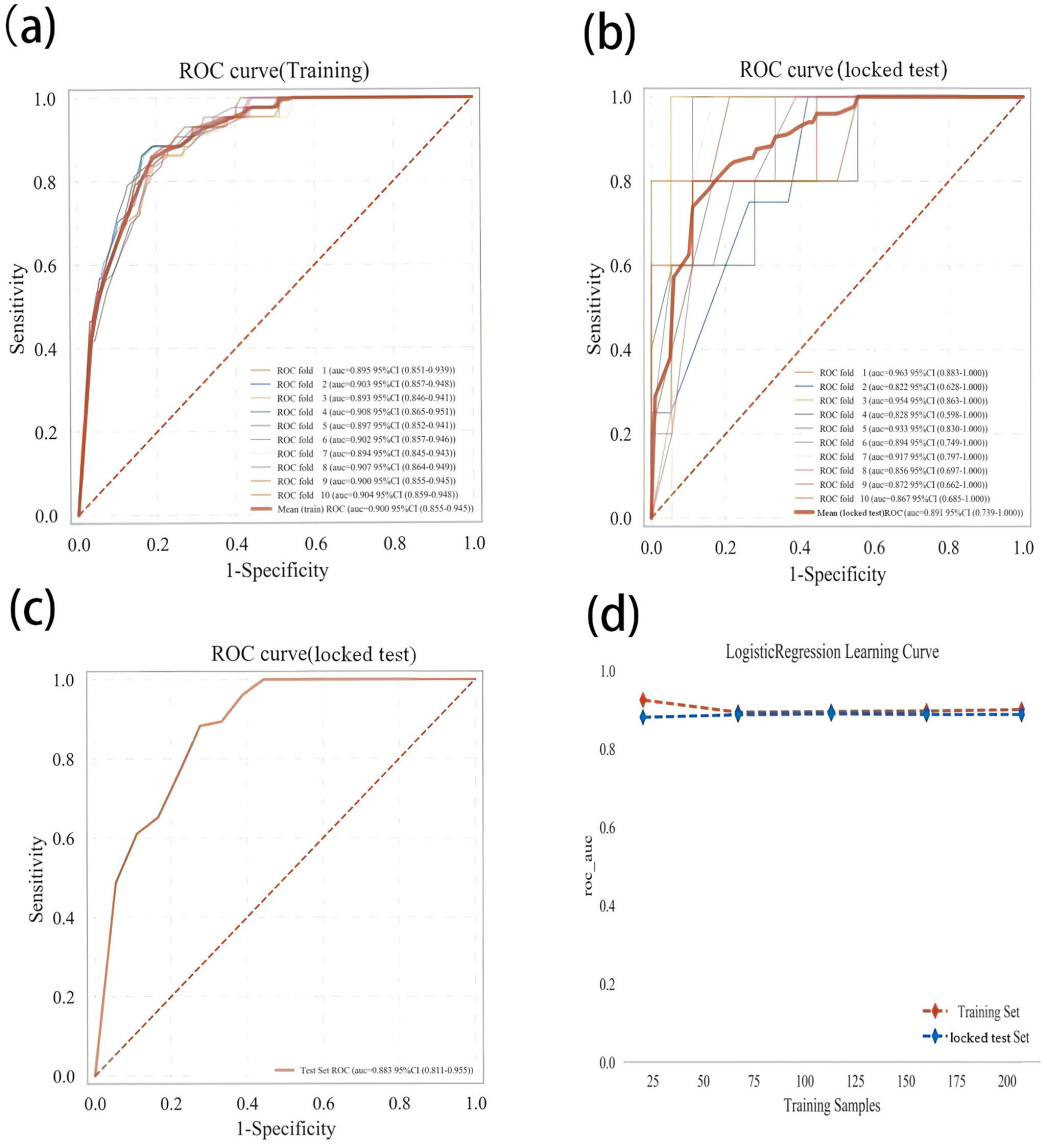
Logistic model training, validation and testing. (a) Training set receiver operating characteristic (ROC) and area under the curve (AUC). (b) Locked test‐set ROC and AUC. Trained and cross‐validated using 10% of patients with aneurysmal subarachnoid hemorrhage (aSAH); solid lines of different colors represent 10 outcomes. (c) Locked test set ROC and AUC. Results obtained using 30% of patients with aSAH. (d) Learning curve. Red dashed lines indicate the training set; blue dashed lines indicate the locked test set. Values are presented as mean ± 95% CI.

### 3.7 Visualizing Model Interpretation Using Shapley Additive Explanations

To interpret the selected variables visually, SHAP analysis was employed to illustrate how these variables predict the development of postoperative hydrocephalus following aSAH. Figure 5a displays the four most important features in the model. Within each feature importance line, points of varying colors represent the attribution of outcomes across all patients, with red indicating high‐risk values and blue indicating low‐risk values. Factors such as combined IVH and absence of intraoperative lateral ventriculostomy drainage promote postoperative hydrocephalus formation in patients with aSAH. Figure 5b displays the ranking of the four risk factors evaluated by the average absolute SHAP value, where the SHAP value on the x‐axis indicates the importance of the predictive model. Additionally, a typical example is provided to illustrate the model's interpretability: patients who developed postoperative hydrocephalus had SHAP prediction scores of 0.81 (Figure 5c) and 0.7 (Figure 5d).

**FIGURE 5:**
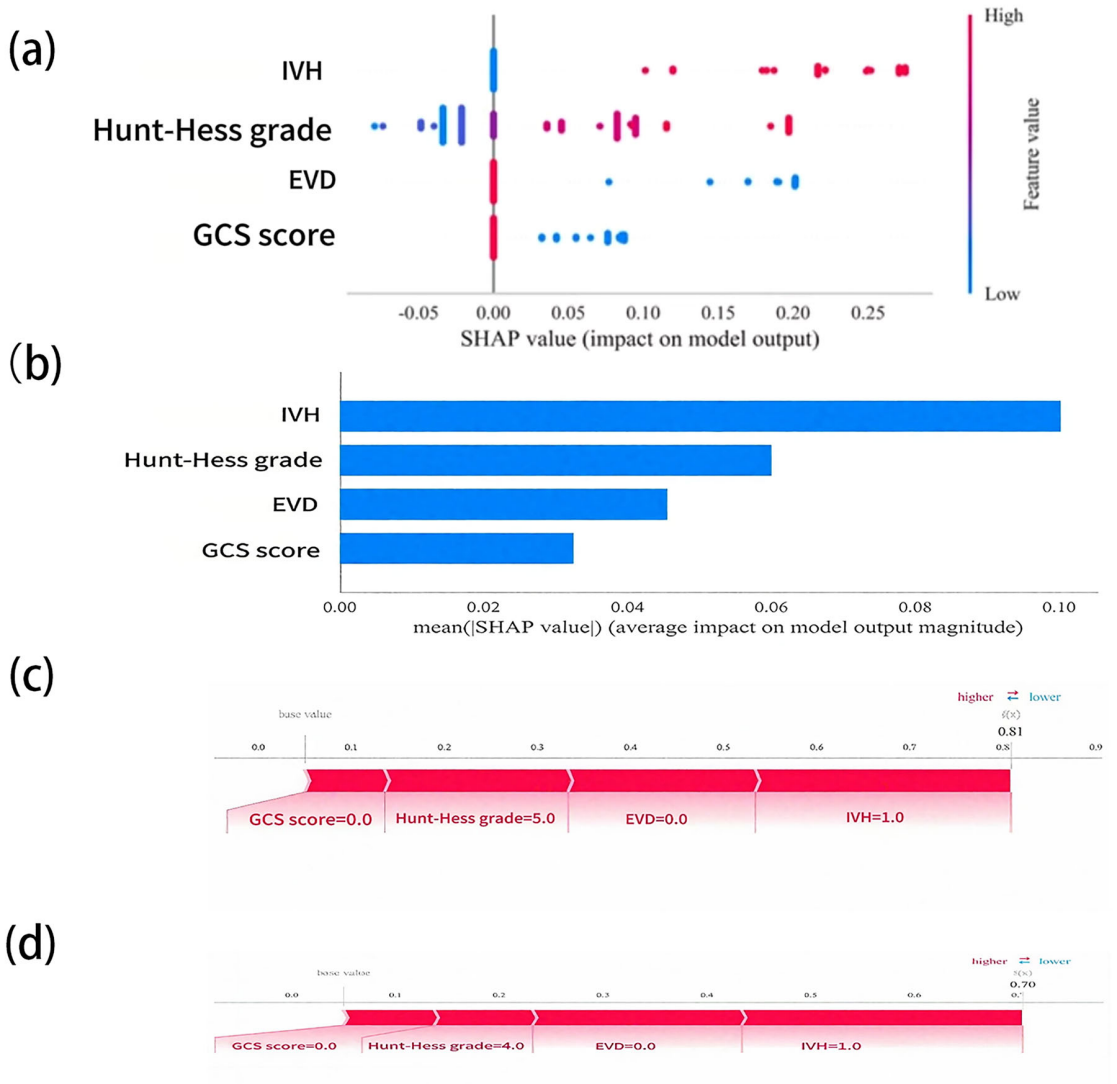
Shapley additive explanations (SHAP) interpretation model. (a) Attributes of SHAP features. Each line represents a feature, with the x‐axis showing SHAP values. Red dots indicate higher feature values; blue dots indicate lower values. (b) Feature importance ranking based on SHAP. The matrix plot illustrates the importance of each covariate in developing the final predictive model. (c) Feature values corresponding to postoperative hydrocephalus in patients without intraoperative lateral ventriculostomy and without concomitant intraventricular hemorrhage (IVH). Postoperative lateral ventriculostomy status = 0 indicates absence of the procedure; concomitant IVH status = 1 indicates its presence. The SHAP values represent individual patient prediction features and their contribution to the prediction of postoperative hydrocephalus. Bolded numbers denote probability predictions (f[x]), and baseline values represent predictions without model input; f(x) is the log‐ratio for each observation. Red features indicate an increased predicted risk of postoperative hydrocephalus; blue features indicate a reduced predicted risk of postoperative hydrocephalus. Arrow length aids visualization of prediction influence‐longer arrows denotes greater impact. (d) SHAP force plot for another representative patient, showing a predicted probability of 0.70. The presence of IVH, Hunt–Hess grade 4, absence of EVD, and the standardized GCS score collectively shifted the model prediction toward a higher risk of postoperative hydrocephalus. Red features indicate factors that increase the predicted risk, whereas blue features indicate factors that decrease the predicted risk.

To further assess the potential risk of overfitting, the events‐per‐variable ratio was reported, and a bootstrap optimism correction was applied. Among the 331 included patients, 66 developed postoperative hydrocephalus, and approximately 45 outcome events were available in the training set. Because the final logistic regression model included four variables, the events‐per‐variable ratio was approximately 11.3 in the training set and 16.5 in the full cohort. Bootstrap internal validation with 1,000 resamples showed an apparent AUC of 0.902, a mean optimism of 0.022, and an optimism‐corrected AUC of 0.880. The optimism‐corrected accuracy, sensitivity, specificity, positive predictive value, negative predictive value, F1 score and Brier score were 0.710, 0.858, 0.677, 0.376, 0.961, 0.523 and 0.141, respectively (Table S7). These findings suggest that although some optimism was present, the corrected model performance remained acceptable. Nevertheless, given the limited number of outcome events, the comparison of eight ML algorithms should be interpreted as exploratory.

### 3.8 Sensitivity Analysis Using Admission Glasgow Coma Scale Scores

A sensitivity analysis using the admission GCS score as a continuous variable showed results comparable with those of the primary dichotomized model. In the locked test set, the continuous‐GCS model achieved an AUC of 0.887, an accuracy of 0.720, a sensitivity of 0.861, a specificity of 0.695, a positive predictive value of 0.392, a negative predictive value of 0.955, an F1 score of 0.538, and a Brier score of 0.139. The calibration slope was 0.91, and the calibration intercept was −0.04. These findings were similar to those of the primary model using the GCS cutoff of 11, suggesting that the main findings were not materially driven by dichotomization of GCS score. Therefore, the dichotomized model was retained as the primary model because of its clinical interpretability and bedside applicability (Table S8).

## 4 Discussion

This study employed traditional regression and ML methods to develop and validate a predictive model for postoperative hydrocephalus following aSAH. Based on cohort data from 331 patients, we identified the absence of EVD and concomitant IVH as significant predictors. Compared with more complex ML algorithms, the logistic regression model showed stable discrimination, better calibration, and greater interpretability in this dataset. These findings are consistent with, and build upon, prior research that has underscored the complexity of predicting this common complication (Wilson et al. 2017). From a clinical perspective, the model is best understood as a postoperative monitoring triage tool. Its high negative predictive value suggests potential usefulness for identifying patients at low risk who may be suitable for routine surveillance, whereas its modest positive predictive value indicates that high‐risk predictions should be interpreted cautiously. Patients who are model positive should receive closer observation and timely reassessment rather than immediate diagnosis or automatic therapeutic intervention.

Low GCS scores often accompany autonomic dysfunction, affecting CSF dynamics (Rao et al. 2024; Mistry et al. 2023). Deng et al. found that each 1‐point decrease in GCS increases the risk of hydrocephalus by 18% (Deng et al. 2021), consistent with the findings of this study. A high Hunt–Hess score is another predictor, with hydrocephalus occurring in 30%–50% of patients scoring ≥IV on this scale (Li et al. 2024). This score comprehensively assesses the severity of neurological deficits, with higher scores indicating diffuse vasospasm, cerebral hypoperfusion. and blood–brain barrier disruption (Hayashi et al. 2021). These pathological changes promote fibrin exudation and arachnoid granule adhesion, ultimately obstructing CSF drainage (Daou et al. 2021). A cohort study involving 580 patients demonstrated that patients with Hunt–Hess IV–V grade had a 3.2‐fold higher risk of hydrocephalus than those with a lower grade (Vergouwen et al. 2010). The association between intraoperative EVD and postoperative hydrocephalus should be interpreted cautiously because of temporal and causal ambiguity. External ventricular drainage is a clinician‐selected intervention and is more likely to be used in patients with more severe hemorrhage, impaired consciousness, acute ventricular enlargement, or IVH. Therefore, the absence or presence of EVD should not be interpreted as directly causing an increase or decrease in hydrocephalus risk. Rather, EVD may partly reflect baseline disease severity, treatment selection, and early postoperative clinical status. In the present model, EVD was included as a perioperative clinical variable for immediate postoperative risk stratification, not as a causal exposure. Although admission GCS score, Hunt–Hess grade, and IVH were included in the final model to partly account for disease severity, residual confounding by indication cannot be excluded in this retrospective cohort (Wu et al. 2023). Previous studies have reported associations between CSF drainage strategies and the subsequent risk of hydrocephalus following aSAH (Rincon et al. 2010). Its protective mechanisms include clearing ventricular haematoma, reducing fibrin deposition, and blocking arachnoid villi fibrosis (Miller et al. 2014). Notably, EVD timing and drainage methods influence efficacy; early drainage (<24 h) combined with a stepwise clamping strategy further reduces risk (Capion et al. 2024). This study emphasizes the importance of intraoperative EVD, consistent with Musmar's theory (Musmar et al. 2025). The study found that concurrent IVH is one of the strongest predictors of postoperative hydrocephalus, largely consistent with previous findings (Lenkeit et al. 2024). Intraventricular hemorrhage directly obstructs the foramen of Monro, the cerebral aqueduct, and the fourth ventricle outlet, causing acute obstructive hydrocephalus (Osgood 2021). Simultaneously, degradation products of red blood cells (e.g., hemoglobin, iron ions) trigger inflammatory responses leading to ependymal ciliary dysfunction and chronic fibrosis (Lee 2025). Studies demonstrate a strong association between IVH and hydrocephalus (Roeder et al. 2019). Intraventricular hemorrhage may contribute to postoperative hydrocephalus through several biologically plausible mechanisms, including obstruction of CSF pathways, inflammatory responses triggered by blood degradation products, ependymal injury, impaired ciliary function, and fibrosis affecting CSF circulation. When IVH coexists with SAH, these processes may further aggravate impairment of CSF absorption and circulation (Xu et al. 2024). These findings differ from some previous studies in which age, modified Fisher grade, acute hydrocephalus, and treatment modality were reported as significant predictors of chronic or shunt‐dependent hydrocephalus. Such discrepancies may be related to differences in outcome definition, follow‐up duration, variable selection methods, and patient populations (Ziai et al. 2014). In our cohort, the outcome was short‐term postoperative hydrocephalus within 3 weeks, whereas many previous studies focused on chronic or shunt‐dependent hydrocephalus. In addition, IVH and severity scores may have captured part of the risk represented by Fisher grade or other hemorrhage‐related variables, thereby weakening their independent effects in multivariable modelling. A possible explanation is that these factors are closely associated with IVH itself; when IVH is included in the analysis, the effects of other factors become less pronounced. Similarly, patients with more severe conditions are typically more likely to undergo EVD, thereby reducing their perceived risk (Zhao et al. 2020). This underscores the importance of multivariate analysis. Biochemical markers in CSF (Pro, Glu, Cl) and fibrinogen in blood showed no significant association in this study. This may relate to differing testing time points or insufficient sample sizes. Recent studies suggest that inflammatory markers and coagulation‐related substances in CSF may be more strongly associated with chronic hydrocephalus, though their predictive value for acute hydrocephalus requires further investigation (Daou et al. 2021). Fibrinogen may influence hydrocephalus by promoting coagulation and inflammation, although evidence remains inconclusive (Li et al. 2019). Treatment modality for aneurysms in this study (craniotomy clipping or endovascular embolization) did not significantly affect hydrocephalus risk. This finding aligns with most recent studies (Prasad et al. 2023). Modern treatments are relatively safe, with no apparent difference in CSF circulation between the two approaches (Hoh et al. 2023). However, some studies suggest that open cranial clipping should be preferred for patients with Hunt–Hess III–V aSAH (Yang et al. 2021). Compared with endovascular intervention, open cranial clipping allows intraoperative evacuation of intracerebral and ventricular hematomas, preventing obstructive hydrocephalus by restoring open ventricular circulation (de Oliveira Manoel et al. 2025; Iqbal et al. 2024 Dec).

Several recent studies have focused on the prediction of chronic or shunt‐dependent hydrocephalus after aSAH. Zhang et al. developed a retrospective prediction model for chronic hydrocephalus following aSAH, supporting the feasibility of individualised risk assessment in this population (Zhang et al. 2023). More recently, Hajikarimloo et al. systematically reviewed and meta‐analyzed ML models for predicting chronic or shunt‐dependent hydrocephalus after SAH, showing that ML‐based approaches have promising predictive performance but still require further standardization and external validation (2026). Islamagič et al. developed the IS‐Score, a novel predictive model for shunt requirement after aSAH, highlighting the clinical importance of simple and interpretable scoring systems (2026). In addition, the 2023 American Heart Association and American Stroke Association guidelines emphasize evidence‐based monitoring and management of aSAH‐related complications. Compared with these studies, our model focuses on early postoperative hydrocephalus rather than chronic or shunt‐dependent hydrocephalus and incorporates SHAP analysis to improve interpretability. However, differences in outcome definition, prediction timing, candidate variables, and validation strategy may partly explain the variability among different models (Abdulazim et al. 2023). Machine‐learning algorithms offer flexibility in handling high‐dimensional data and complex interactions (Steyerberg et al. 2013). In this study, tree‐based models, such as XGBoost, exhibited high training set AUC but degraded locked test‐set performance, suggesting overfitting. In contrast, logistic regression delivered robust and interpretable results, supporting its application in clinical prediction models with limited sample sizes (Deo 2015).

This study has several limitations. First, the principal limitation of this study is the absence of external validation. This was a single‐center retrospective study, and the outcome distribution was imbalanced, with postoperative hydrocephalus occurring in 66 of 331 patients. Although a Locked test set was used to provide an internal estimate of model performance, this design cannot establish generalizability across different centers, patient populations, imaging protocols, neurosurgical practices, or postoperative monitoring strategies. Therefore, the present model should be regarded as an internally developed early postoperative risk stratification tool rather than a fully validated clinical prediction model. Prospective multicenter external validation is required before clinical implementation. Second, missing data represent another limitation. Postoperative CSF Cl, Glu, and Pro levels had 13.3% missingness. Although these variables were imputed using a leakage‐aware KNN approach and were not retained in the final model, residual bias related to missing postoperative CSF measurements cannot be completely excluded. We did not perform a complete‐case model because excluding patients with missing CSF variables would further reduce the number of outcome events and increase model instability in this event‐limited cohort. Future prospective studies should standardize postoperative CSF testing protocols to reduce missingness and allow more robust sensitivity analyses. Third, despite imputation of missing data, potential biases may persist. Fourth, the study focused solely on short‐term (within 3 weeks) hydrocephalus occurrence, with long‐term outcomes requiring further investigation. In particular, EVD placement may be subject to confounding by indication, as patients receiving EVD are usually more severely affected at baseline. Although severity‐related variables were included in the model to partially adjust for this issue, the retrospective design precluded full control of treatment‐selection bias. Future prospective studies with standardized EVD indications or propensity score‐based adjustment are needed to clarify the independent effect of EVD further. To reduce confounding related to disease severity, EVD was included in the final model alongside admission GCS score, Hunt–Hess grade, and IVH, rather than as an isolated causal predictor. In addition, we acknowledge that dichotomization of continuous variables may reduce information and statistical efficiency. In this study, dichotomization was used primarily to improve clinical interpretability and bedside applicability. The cut‐off values were derived from the training set and internally assessed using bootstrap resampling. Nevertheless, continuous modelling or flexible approaches, such as restricted cubic splines, may better preserve prognostic information and should be evaluated in future external validation studies.

## 5 Conclusion

This study developed an early postoperative risk‐stratification model for hydrocephalus following aSAH. Admission GCS score, intraoperative EVD/lateral ventricular drainage, and IVH were the main contributors to model prediction, whereas Hunt–Hess grade was retained as a severity‐related covariate with limited independent contribution. The logistic regression model showed favorable discrimination and a high negative predictive value in the locked test set. However, because the positive predictive value was modest, the model should be used to support postoperative monitoring triage, repeated imaging decisions, and timely clinical reassessment rather than as a standalone diagnostic or preventive tool.

## Author Contributions

Conception and design of the work: Li F G, Fu K X; Data collection: Zhou S F, Gong Z P, Du C Y, Zhao Y X, Yang W L, Zhang C Y, Zhou W K, Li W C; Supervision: Li F G, Fu K X; Analysis and interpretation of the data: Zhou S F, Gong Z P, Du C Y, Zhao Y X, Yang W L, Zhang C Y, Zhou W K, Li W C; Statistical analysis: Li F G, Fu K X, Zhou W K, Li W C; Drafting the manuscript: Li F G, Fu K X; Critical revision of the manuscript: all authors; Approval of the final manuscript: all authors.

## Funding

This work was funded by the 2024 Henan Medical Science and Technology Key Project (No. LHGJ20240486).

## Ethics Statement

This study was conducted in accordance with the Declaration of Helsinki and was approved by the Ethics Committee of the First Affiliated Hospital of Henan Medical University (Approval Number: EC‐025‐10). The ethics committee waived the requirement for written informed consent because this was a retrospective study using de‐identified clinical data.

## Conflicts of Interest

The authors declare no conflicts of interest.

## Supporting information




**Supplementary Material**: brb371869‐sup‐0001‐SuppMat.docxS

## Data Availability

The datasets generated and analyzed during the current study are not publicly available because they contain patient‐level clinical information, but de‐identified data may be made available from the corresponding author upon reasonable request and with approval from the institutional ethics committee. The analysis code and model specification can be provided upon reasonable request to allow reproducibility of the reported results.

## References

[brb371869-bib-0001] Abdulazim, A. , M. Heilig , G. Rinkel , et al. 2023. “Diagnosis of Delayed Cerebral Ischemia in Patients With Aneurysmal Subarachnoid Hemorrhage and Triggers for Intervention.” Neurocritical Care 39, no. 2: 311–319. 10.1007/s12028-023-01812-3. PMC10542310 37537496

[brb371869-bib-0002] Adams, S. T. , and S. H. Leveson . 2012. “Clinical Prediction Rules.” BMJ 344: d8312. 10.1136/bmj.d8312. 22250218

[brb371869-bib-0003] Bazoukis, G. , J. Hall , J. Loscalzo , et al. 2022. “The Inclusion of Augmented Intelligence in Medicine: A Framework for Successful Implementation.” Cell Reports Medicine 3, no. 1: 100485. 10.1016/j.xcrm.2021.100485. PMC8784713 35106506

[brb371869-bib-0004] Belkin, M. , D. Hsu , S. Ma , et al. 2019. “Reconciling Modern Machine‐Learning Practice and the Classical Bias‐Variance Trade‐Off.” Proceedings of the National Academy of Sciences 116, no. 32: 15849–15854. 10.1073/pnas.1903070116. PMC6689936 31341078

[brb371869-bib-0005] Capion, T. , A. Lilja‐Cyron , M. Juhler , et al. 2024. “Prompt Closure versus Gradual Weaning of External Ventricular Drain for Hydrocephalus Following Aneurysmal Subarachnoid Haemorrhage: A Statistical Analysis Plan for the DRAIN Randomised Clinical Trial.” Trials 25, no. 1: 479. 10.1186/s13063-024-08305-4. PMC11251380 39010208

[brb371869-bib-0006] Claassen, J. , and S. Park . 2022. “Spontaneous Subarachnoid Haemorrhage.” Lancet 400, no. 10355: 846–862. 10.1016/S0140-6736(22)00938-2. PMC9987649 35985353

[brb371869-bib-0007] Culebras, D. , L. Pedrosa , A. Mosteiro , et al. 2025. “Prognostic Factors in Aneurysmal Subarachnoid Hemorrhage With Poor Initial Clinical Grade.” Frontiers in Neurology 16: 1536643. 10.3389/fneur.2025.1536643. PMC12000015 40242616

[brb371869-bib-0008] Daou, B. J. , S. S. S. Khalsa , S. K. Anand , et al. 2021. “Volumetric Quantification of Aneurysmal Subarachnoid Hemorrhage Independently Predicts Hydrocephalus and Seizures.” Journal of Neurosurgery 135, no. 4: 1155–1163. 10.3171/2020.8.JNS201273. 33545677

[brb371869-bib-0009] de Hond, A. A. H. , A. M. Leeuwenberg , L. Hooft , et al. 2022. “Guidelines and Quality Criteria for Artificial Intelligence‐Based Prediction Models in Healthcare: A Scoping Review.” NPJ Digital Medicine 5, no. 1: 2. 10.1038/s41746-021-00549-7. PMC8748878 35013569

[brb371869-bib-0010] Deng, H. , E. Goldschmidt , E. Nwachuku , et al. 2021. “Hydrocephalus and Cerebrospinal Fluid Analysis following Severe Traumatic Brain Injury: Evaluation of a Prospective Cohort.” Neurology International 13, no. 4: 527–534. 10.3390/neurolint13040052. PMC8544497 34698266

[brb371869-bib-0011] Deo, R. C. 2015. “Machine Learning in Medicine.” Circulation 132, no. 20: 1920–1930. 10.1161/CIRCULATIONAHA.115.001593. PMC5831252 26572668

[brb371869-bib-0012] de Oliveira Manoel, A. L. , F. G. Zampieri , A. Goffi , et al. 2026. “Early Motor Glasgow Coma Scale Predicts Unfavorable Functional Outcome after Poor‐Grade Subarachnoid Hemorrhage.” Canadian Journal of Neurological Sciences 53, no. 4: 570–576. 10.1017/cjn.2025.10373. 40702663

[brb371869-bib-0013] Dupont, S. , and A. A. Rabinstein . 2013. “CT Evaluation of Lateral Ventricular Dilatation After Subarachnoid Hemorrhage: Baseline Bicaudate Index Values [correction of balues].” Neurological Research 35, no. 2: 103–106. 10.1179/1743132812Y.0000000121. 23336389

[brb371869-bib-0014] Eide, P. K. , A. H. Pripp , and G. Ringstad . 2020. “Magnetic Resonance Imaging Biomarkers of Cerebrospinal Fluid Tracer Dynamics in Idiopathic Normal Pressure Hydrocephalus.” Brain Communications 2, no. 2: fcaa187. 10.1093/braincomms/fcaa187. PMC7753057 33381757

[brb371869-bib-0015] Greener, J. G. , S. M. Kandathil , L. Moffat , et al. 2022. “A Guide to Machine Learning for Biologists.” Nature Reviews Molecular Cell Biology 23, no. 1: 40–55. 10.1038/s41580-021-00407-0. 34518686

[brb371869-bib-0016] Hajikarimloo, B. , I. Mohammadzadeh , M. A. Habibi , et al. 2026. “Machine Learning Models in the Prediction of Chronic or Shunt‐Dependent Hydrocephalus Following Subarachnoid Hemorrhage: A Systematic Review and Meta‐Analysis.” Neuroradiology Journal 39, no. 1: 31–41. 10.1177/19714009251345104. PMC12102083 40405362

[brb371869-bib-0017] Hayashi, K. , Y. Ogawa , T. Fujimoto , et al. 2021. “Vasospasm and Hydrocephalus Following Subarachnoid Hemorrhage Are Less Frequent in Coil Embolization Than in Clipping.” Journal of Neuroendovascular Therapy 15, no. 4: 201–206. 10.5797/jnet.oa.2020-0130. PMC10370927 37501688

[brb371869-bib-0018] Hoh, B. L. , N. U. Ko , S. Amin‐Hanjani , et al. 2023. “Guideline for the Management of Patients with Aneurysmal Subarachnoid Hemorrhage: A Guideline from the American Heart Association/American Stroke Association.” Stroke; A Journal of Cerebral Circulation 54, no. 7: e314–e370. 10.1161/STR.0000000000000436. 37212182

[brb371869-bib-0019] Iqbal, I. K. , N. Ansari , M. Subhan , et al. 2024 Dec. “Outcomes of Fenestration of Lamina Terminalis for Hydrocephalus Following Clipping of Ruptured Aneurysms of Anterior Circulation.” Supplement, Pakistan Journal of Medical Sciences 40, no. (PINS Suppl) 12: S37–S40. 10.12669/pjms.40.12(PINS).11122. PMC11654651 39703961

[brb371869-bib-0020] Islamagič, L. , I. Djebbara , T. H. Nielsen , et al. 2026. “Development of the IS‐Score: A Novel Predictive Model for Shunt Requirement Following Aneurysmal Subarachnoid Hemorrhage.” Neurocritical Care 45, no. 1: 237–246. 10.1007/s12028-026-02461-y. PMC13369536 41792522

[brb371869-bib-0021] Lee, T. H. 2025. “Intracerebral Hemorrhage.” Cerebrovascular Diseases Extra 15, no. 1: 1–8. 10.1159/000542566. PMC11658787 39557033

[brb371869-bib-0022] Lenkeit, A. , M. D. Oppong , T. F. Dinger , et al. 2024. “Risk Factors for Poor Outcome After Aneurysmal Subarachnoid Hemorrhage in Patients With Initial Favorable Neurological Status.” Acta Neurochirurgica 166, no. 1: 93. 10.1007/s00701-024-05968-5. PMC10879324 38376665

[brb371869-bib-0023] Li, K. , C. D. Barras , R. V. Chandra , et al. 2019. “A Review of the Management of Cerebral Vasospasm after Aneurysmal Subarachnoid Hemorrhage.” World Neurosurgery 126: 513–527. 10.1016/j.wneu.2019.03.083. 30898740

[brb371869-bib-0024] Li, X. , Y. Wang , B. Xia , et al. 2024. “Lumboperitoneal Shunt and Ventriculoperitoneal Shunt for Chronic Hydrocephalus After Aneurysmal Subarachnoid Hemorrhage: A Comparison.” Frontiers in Surgery 11: 1368493. 10.3389/fsurg.2024.1368493. PMC10963422 38533091

[brb371869-bib-0025] Lundberg, S. M. , G. Erion , H. Chen , et al. 2020. “From Local Explanations to Global Understanding With Explainable AI for Trees.” Nature Machine Intelligence 2, no. 1: 56–67. 10.1038/s42256-019-0138-9. PMC7326367 32607472

[brb371869-bib-0026] Miller, B. A. , N. Turan , M. Chau , et al. 2014. “Inflammation, Vasospasm, and Brain Injury After Subarachnoid Hemorrhage.” BioMed Research International 2014: 384342. 10.1155/2014/384342. PMC4106062 25105123

[brb371869-bib-0027] Mistry, A. M. , J. Naidugari , M. J. Feldman , et al. 2023. “Impact of Fludrocortisone on the Outcomes of Subarachnoid Hemorrhage Patients: A Retrospective Analysis.” Preprint, medRxiv, September 28. 10.1101/2023.09.28.23296246. 38387759

[brb371869-bib-0028] Muschelli, J. 2020. “ROC and AUC With a Binary Predictor: A Potentially Misleading Metric.” Journal of Classification 37, no. 3: 696–708. 10.1007/s00357-019-09345-1. PMC7695228 33250548

[brb371869-bib-0029] Musmar, B. , H. Abdalrazeq , J. M. Roy , et al. 2025. “Outcomes of External Ventricular Drainage and Lumbar Drainage in Aneurysmal Subarachnoid Hemorrhage: A Systematic Review and Meta‐Analysis.” International Journal of Stroke 20: 17474930251361211. 10.1177/17474930251361211. 40662333

[brb371869-bib-0030] Obuchowski, N. A. , and J. A. Bullen . 2018. “Receiver Operating Characteristic (ROC) Curves: Review of Methods With Applications in Diagnostic Medicine.” Physics in Medicine and Biology 63, no. 7: 07TR01. 10.1088/1361-6560/aab4b1. 29512515

[brb371869-bib-0031] Osgood, M. L. 2021. “Aneurysmal Subarachnoid Hemorrhage: Review of the Pathophysiology and Management Strategies.” Current Neurology and Neuroscience Reports 21, no. 9: 50. 10.1007/s11910-021-01136-9. 34308493

[brb371869-bib-0032] Padula, W. V. , D. G. Armstrong , P. J. Pronovost , et al. 2024. “Predicting Pressure Injury Risk in Hospitalised Patients Using Machine Learning With Electronic Health Records: A US Multilevel Cohort Study.” BMJ Open 14, no. 4: e082540. 10.1136/bmjopen-2023-082540. PMC11146395 38594078

[brb371869-bib-0033] Panciani, P. P. , G. Palandri , G. Petrella , et al. 2025. “Idiopathic Normal Pressure Hydrocephalus: A Systematic Review and a Streamlined Six‐Step Algorithm Endorsed by the Italian Society of Neurosurgery (SINCH).” Journal of Neurosurgical Sciences 69, no. 1: 92–101. 10.23736/S0390-5616.25.06429-X. 40045807

[brb371869-bib-0034] Prasad, M. K. , S. Marandi , B. Mishra , et al. 2023. “Association of Fibrinogen with Ischemic Stroke: A Systematic Review and Meta‐Analysis.” Cureus 15, no. 1: e34335. 10.7759/cureus.34335. PMC9884496 36721710

[brb371869-bib-0035] Rao, D. , L. Yang , X. Enxi , et al. 2024. “A Predictive Model in Patients With Chronic Hydrocephalus Following Aneurysmal Subarachnoid Hemorrhage: A Retrospective Cohort Study.” Frontiers in Neurology 15: 1366306. 10.3389/fneur.2024.1366306. PMC11137279 38817542

[brb371869-bib-0036] Rincon, F. , E. Gordon , R. M. Starke , et al. 2010. “Predictors of Long‐Term Shunt‐Dependent Hydrocephalus After Aneurysmal Subarachnoid Hemorrhage. Clinical Article.” Journal of Neurosurgery 113, no. 4: 774–780. 10.3171/2010.2.JNS09376. 20367072

[brb371869-bib-0037] Roeder, S. S. , M. I. Sprügel , J. A. Sembill , et al. 2019. “Influence of the Extent of Intraventricular Hemorrhage on Functional Outcome and Mortality in Intracerebral Hemorrhage.” Cerebrovascular Diseases 47, no. 5–6: 245–252. 10.1159/000501027. 31212293

[brb371869-bib-0038] Sauerbrei, W. , E. Kipruto , and J. Balmford . 2023. “Effects of Influential Points and Sample Size on the Selection and Replicability of Multivariable Fractional Polynomial Models.” Diagnostic and Prognostic Research 7, no. 1: 7. 10.1186/s41512-023-00145-1. PMC10111698 37069621

[brb371869-bib-0039] Shortliffe, E. H. , and M. J. Sepúlveda . 2018. “Clinical Decision Support in the Era of Artificial Intelligence.” JAMA 320, no. 21: 2199–2200. 10.1001/jama.2018.17163. 30398550

[brb371869-bib-0040] Steyerberg, E. W. , K. G. Moons , and D. A. van der Windt , et al. 2013. “Prognosis Research Strategy (PROGRESS) 3: Prognostic Model Research.” Plos Medicine 10, no. 2: e1001381. 10.1371/journal.pmed.1001381. PMC3564751 23393430

[brb371869-bib-0041] Sweeney, K. , N. Silver , and M. Javadpour . 2016. “Subarachnoid Haemorrhage (spontaneous aneurysmal).” BMJ Clinical Evidence 2016: 1213. PMC4794735 26983641

[brb371869-bib-0042] Vayena, E. , A. Blasimme , and I. G. Cohen . 2018. “Machine Learning in Medicine: Addressing Ethical Challenges.” Plos Medicine 15, no. 11: e1002689. 10.1371/journal.pmed.1002689. PMC6219763 30399149

[brb371869-bib-0043] Vergouwen, M. D. , M. Vermeulen , J. van Gijn , et al. 2010. “Definition of Delayed Cerebral Ischemia After Aneurysmal Subarachnoid Hemorrhage as an Outcome Event in Clinical Trials and Observational Studies: Proposal of a Multidisciplinary Research Group.” Stroke; A Journal of Cerebral Circulation 41, no. 10: 2391–2395. 10.1161/STROKEAHA.110.589275. 20798370

[brb371869-bib-0044] Wilson, C. D. , S. Safavi‐Abbasi , H. Sun , et al. 2017. “Meta‐Analysis and Systematic Review of Risk Factors for Shunt Dependency After Aneurysmal Subarachnoid Hemorrhage.” Journal of Neurosurgery 126, no. 2: 586–595. 10.3171/2015.11.JNS152094. 27035169

[brb371869-bib-0045] Wu, B. , Y. Zhou , H. Fan , et al. 2023. “Cerebrospinal Fluid Drainage and Chronic Hydrocephalus in Aneurysmal Subarachnoid Hemorrhage Patients With Intraventricular Hemorrhage.” Frontiers in Neurology 14: 1302622. 10.3389/fneur.2023.1302622. PMC10758233 38164202

[brb371869-bib-0046] Xu, X. , H. Yang , X. Wang , et al. 2024. “Effect of External Ventricular Drainage on Shunt‐Dependent Hydrocephalus and Prognosis After Microsurgical Clipping in Patients With Poor‐Grade Aneurysmal Subarachnoid Hemorrhage.” World Neurosurgery 191: e594–e606. 10.1016/j.wneu.2024.09.004. 39270793

[brb371869-bib-0047] Yang, Y. C. , C. H. Yin , K. T. Chen , et al. 2021. “Prognostic Nomogram of Predictors for Shunt‐Dependent Hydrocephalus in Patients With Aneurysmal Subarachnoid Hemorrhage Receiving External Ventricular Drain Insertion: A Single‐Center Experience and Narrative Review.” World Neurosurgery 150: e12–e22. 10.1016/j.wneu.2021.01.138. 33556600

[brb371869-bib-0048] Zhang, F. , X. F. Cai , W. Zhao , et al. 2023. “A Predictive Model for Chronic Hydrocephalus After Clipping Aneurysmal Subarachnoid Hemorrhage.” Journal of Craniofacial Surgery 34, no. 2: 680–683. 10.1097/SCS.0000000000009036. 36168119

[brb371869-bib-0049] Zhao, D. D. , Z. D. Guo , S. He , et al. 2020. “High Intracranial Pressure May be the Initial Inducer of Elevated Plasma D‐dimer Level After Aneurysmal Subarachnoid Haemorrhage.” International Journal of Neuroscience 130, no. 7: 694–699. 10.1080/00207454.2019.1702546. 31852390

[brb371869-bib-0050] Ziai, W. C. , S. Tuhrim , K. Lane , et al. 2014. “A Multicenter, Randomized, Double‐Blinded, Placebo‐Controlled Phase III Study of Clot Lysis Evaluation of Accelerated Resolution of Intraventricular Hemorrhage (CLEAR III).” International Journal of Stroke 9, no. 4: 536–542. 10.1111/ijs.12097. PMC6931254 24033910

