# Supplementary material for: Development and Validation of an Early Postoperative Risk Stratification Model for Hydrocephalus Following Aneurysmal Subarachnoid Hemorrhage
Source: Brain Behav. 2026 Oct 28;16(11):e71869. doi: 10.1002/brb3.71869 (PMC13715075; doi:10.1002/brb3.71869)
Supplement: Supplementary file 1 [file BRB3-16-e71869-s001.docx]

**Development and Validation of an Early Postoperative Risk Stratification Model for Hydrocephalus Following Aneurysmal Subarachnoid Hemorrhage**

Running title: Postoperative Hydrocephalus Following Aneurysmal Subarachnoid Hemorrhage

**Supporting methods:**

**Patient Grouping and Diagnosis for Aneurysmal Subarachnoid Hemorrhage**

Hydrocephalus primarily manifests as symptoms such as severe headaches and decreased level of consciousness. On imaging, an Evans index (anterior horn ratio of lateral ventricles) >0.3 serves as an objective indicator. This study primarily investigates acute and subacute hydrocephalus: Cranial CT or MRI reveals rapid ventricular system enlargement, typically objectively defined by an Evans index (anterior horn ratio) >0.3. Additional findings include flattening or disappearance of sulci and gyri, along with vanishing aqueduct. These radiographic features combined with acute clinical symptoms confirm diagnosis[10] . Second, diagnostic criteria for subacute hydrocephalus. Symptoms progress over weeks, including gait instability, cognitive decline, and urinary incontinence. Imaging criteria are crucial: serial CT or MRI scans reveal progressive ventricular enlargement, potentially accompanied by periventricular ependymal edema. Characteristically, T2/FLAIR sequences show uniform, symmetrical patchy hyperintensity-commonly termed the "cap sign" or "periventricular halos." These imaging changes, combined with subacute symptoms, support the diagnosis[11]. Finally, diagnostic criteria for chronic hydrocephalus. Chronic hydrocephalus develops slowly, primarily presenting as a triad: gait disturbance, dementia, and urinary incontinence. Imaging criteria include: markedly enlarged ventricles without significant brain atrophy, narrowed corpus callosum angle (<90°), and periventricular interstitial hydrocephalus. Crucially, other neurodegenerative diseases must be excluded. High-resolution MRI sequences aid in assessing cerebrospinal fluid dynamics [12]. This paper defines postoperative hydrocephalus following aSAH as acute hydrocephalus and subacute hydrocephalus, specifically referring to hydrocephalus occurring within 3 weeks postoperatively.

**Final Assignment**

Using the thresholds determined through the above process, continuous variables were converted into binary variables for subsequent modeling.

First, the 331 patients were randomly divided into a training set (231 cases) and a locked test set (100 cases) at a ratio of 7:3. The locked test set was strictly locked throughout the entire model development process, not participating in any model selection, hyperparameter optimization, or feature selection steps, and was used solely for the final evaluation of model performance. Within the training set, we employed nested cross-validation for model selection and hyperparameter optimization to minimize overfitting and evaluation bias. Considering the approximately 1:4 imbalance between hydrocephalus-positive and negative samples in the dataset, we employed weighted cross-entropy loss functions (applicable to XGBoost, LightGBM, etc.) and/or class weight balancing (class_weight='balanced', applicable to Logistic Regression, SVM, etc.) during model training. These strategies adjusted the model's attention to the minority class, preventing prediction bias toward the majority class. The specific workflow is as follows: Outer Loop (Model Selection): Employ 10-fold cross-validation, further dividing the training set into 10 mutually exclusive subsets. In each iteration, one subset is designated as the locked test set, while the remaining nine serve as training subsets. Inner Loop (Hyperparameter Optimization): Within each outer loop training subset, perform 5-fold cross-validation for hyperparameter tuning. Determine optimal hyperparameter combinations for each model using Grid Search or Random Search. Model Comparison: For each candidate model (including XGBoost, Logistic Regression, LightGBM, Random Forest, AdaBoost, SVM, KNN, and Gaussian Naive Bayes), the average AUC value across the outer 10-fold cross-validation is calculated as the performance evaluation metric. The model with the highest average AUC is selected as the optimal model. Final Training and Testing: The optimal model is trained using the entire training set (231 cases) with hyperparameters determined via inner-layer cross-validation. Final performance evaluation is conducted on the locked test set (100 cases). Multiple metrics are employed for model assessment: Decision curve analysis (DCA) was plotted using R software (rmda 1.6) to assess clinical applicability [13]; calibration curves were plotted using Python to quantify prediction accuracy (reporting Brier score, calibration slope, and intercept) [14]; precision-recall (PR) curves were plotted and average precision (AP) was calculated to supplement model performance evaluation [15]. To enhance model interpretability, we computed variable contributions to predictions using Python (shap 0.39.0), constructed a SHAP global interpretation model, and generated individualized SHAP interpreters for each test sample to assess prediction credibility [16-17].

**LASSO regression coefficient**

The main hyperparameters of all machine-learning models are provided in Table S[X]. Logistic regression used L2 regularization, C = 1.0, solver = lbfgs, max_iter = 100, and tol = 0.0001. Random Forest used 20 trees, the Gini criterion, max_features = sqrt, min_samples_split = 2, and min_samples_leaf = 1. LightGBM used boosting_type = gbdt, learning_rate = 0.1, n_estimators = 100, num_leaves = 31, max_depth = −1, subsample = 1.0, and colsample_bytree = 1.0. AdaBoost used n_estimators = 50 and learning_rate = 1.0. KNN used n_neighbors = 5, weights = uniform, algorithm = auto, and p = 2. SVM used an RBF kernel, C = 1.0, gamma = scale, probability = True, and tol = 0.001. GaussianNB used var_smoothing = 1e−9.

**Statistical Analysis**

Specifically, the K-Nearest Neighbors (KNN) algorithm was employed for imputation. This algorithm identifies the k most similar "neighbors" within the dataset for each missing value based on similarity to all other complete variables, then imputes the missing value using the mean of these neighbors for the variable in question. The K value was set to 5. All imputation operations were performed using the KNNImputer function from Python's scikit-learn library, with a random seed of 42 to ensure reproducibility of results. Following imputation, subsequent normality tests and statistical analyses were performed. The Shapiro-Wilk test assessed data normality. Variables meeting normality requirements were expressed as mean ± standard deviation ($\bar{\text{x}}\text{±}\text{s}$). Non-normally distributed data were presented as median [interquartile range] (IQR), categorical variables should be expressed as frequency (%). $\text{t}$Two groups of normally distributed data were compared using the independent samples t-test (t-test). Two groups of non-normally distributed data were compared using nonparametric tests (Mann-Whitney U test). For comparing categorical variables: When the expected frequency ≥ 5, the chi-square test was used; When 1 ≤ expected frequency ≤ 5, the corrected chi-square test was used; When at least one expected value is less than 1, Fisher's exact probability test was used for variable screening. Variables with$\text{P}$ < 0.15 were included as candidates. These candidate variables were compared across the validation and training sets. P-value < 0.05 was considered statistically significant. ROC analysis was performed using Python's "sklearn 0.22.1" to calculate the AUC and determine the optimal cutoff value. Statistical analysis was performed using R (version 4.0.3). Preliminary variable screening was conducted via LASSO regression using R's "glmnet 4.1.2" package. The "glm" function was applied for binary logistic regression analysis to identify final risk factors. Note that when the independent variable is categorical, the group with the minimum value serves as the reference category. The statistical significance level was set at α=0.05. For model performance metrics, we report point estimates and 95% confidence intervals (95% CI). The confidence interval for the area under the ROC curve (AUC) was calculated using the DeLong method, a theoretically derived standard approach for comparing ROC curves and estimating AUC intervals. For confidence intervals of other performance metrics (e.g., accuracy, sensitivity, specificity), we employed nonparametric Bootstrap resampling. Specifically, 2000 Bootstrap samples with replacement were drawn from the locked test set. Each metric was computed for each sample, and the 95% confidence interval was derived using the percentile method based on the distribution of the 2000 Bootstrap estimates. All Bootstrap analyses were performed with a fixed random seed of 12345 to ensure reproducibility of results.

**Supporting results:**

**Table S1. LASSO Coefficient Table**

| Variable | Coefficient at minimum mean squared error | Coefficient at one-standard-error rule |
| --- | --- | --- |
| Intercept | -2.064 | -0.801 |
| GCS score at admission | -0.497 | -0.496 |
| Hunt-Hess classification at admission | 0.203 | 0.003 |
| Fisher classification at admission | 0.186 | 0.000 |
| Aneurysm size | 0.000 | 0.000 |
| Surgical approach | 0.000 | 0.000 |
| Lamina terminalis fenestration | 0.148 | 0.000 |
| External ventricular drainage | -0.969 | -0.674 |
| Intraventricular hemorrhage | 1.105 | 0.885 |
| Parenchymal hemorrhage | 0.000 | 0.000 |
| Postoperative lumbar puncture | 0.000 | 0.000 |
| Postoperative lumbar cistern drainage | 0.564 | 0.000 |
| Postoperative CSF glucose level | -0.128 | 0.000 |
| Postoperative CSF protein level | 0.000 | 0.000 |

**Table S2. TRIPOD+AI checklist**

| TRIPOD+AI item | Reporting item | Location in manuscript | Comment |
| --- | --- | --- | --- |
| Item 1 | Title and abstract | Title; Abstract | Prediction model and target outcome described |
| Item 2 | Background and objectives | Introduction | Clinical need and study aim described |
| Item 3 | Source of data | Methods, Study population | Single-center retrospective cohort, 2018–2024 |
| Item 4 | Participants | Methods | Inclusion and exclusion criteria described |
| Item 5 | Outcome | Methods | Postoperative hydrocephalus within 3 weeks defined |
| Item 6 | Predictors | Methods | Candidate predictors and final predictors described |
| Item 7 | Sample size | Results | 331 patients, 66 events |
| Item 8 | Missing data | Statistical Analysis | KNN imputation and leakage prevention described |
| Item 9 | Model development | Methods/Results | LASSO, logistic regression, ML models described |
| Item 10 | Model evaluation | Methods/Results | AUC, sensitivity, specificity, PPV, NPV, calibration, DCA |
| Item 11 | Internal validation | Methods/Results | 10-fold cross-validation, locked test set, bootstrap correction |
| Item 12 | Model interpretation | Results | SHAP analysis described |
| Item 13 | Limitations | Discussion | Single-center design, no external validation, imbalance, EVD confounding |
| Item 14 | Clinical use | Discussion/Conclusion | Monitoring triage rather than diagnosis/prevention |
| Item 15 | Transparency | Supplementary Materials | Checklist supplied |

**Table S3. Baseline Characteristics of Training and Test Cohorts**

| Variable | Categorical variable | Test Set 0 (n=231) | Locked Test Set (n=100) | $t/x^{2}$ | p |
| --- | --- | --- | --- | --- | --- |
| Hydrocephalus (Outcome Variable) | Yes | 45 (19.5%) | 20 (20.0%) | 0.012 | 0.912 |
|  | No | 186 (80.5%) | 80 (80.0%) |  |  |
| Hunt-Hess classification at admission | Grade 1 | 47 (20.346) | 19 (19.000) | 1.542 | 0.819 |
|  | Level 2 | 68 (29.437) | 24(24.000) |  |  |
|  | Level 3 | 26 (11.255) | 12 (12.000) |  |  |
|  | Level 4 | 66 (28.571) | 32 (32.000) |  |  |
|  | Level 5 | 24(10.390) | 13 (13.000) |  |  |
| Fisher classification at admission | Grade 1 | 49 (21.212) | 20(20.000) | 0.751 | 0.861 |
|  | Level 2 | 46(19.913) | 17 (17.000) |  |  |
|  | Level 3 | 43 (18.615) | 18 (18.000) |  |  |
|  | Level 4 | 93 (40.260) | 45 (45.000) |  |  |
| Aneurysm size | ≤10mm | 127(54.978) | 65(65.000) | 2.877 | 0.090 |
|  | ＞10mm | 104(45.022) | 35(35.000) |  |  |
| Surgical Approach | Surgical clamping | 105(45.455) | 54(54.000) | 2.042 | 0.153 |
|  | Vascular Intervention | 126(54.545) | 46(46.000) |  |  |
| Intraoperative endoscopic fistulization | No | 189 (81.818) | 82 (82.000) | 0.002 | 0.969 |
|  | Yes | 42(18.182) | 18 (18.000) |  |  |
| Intraoperative EVD | No | 40(17.316) | 24(24.000) | 1.999 | 0.157 |
|  | Yes | 191(82.684) | 76(76.000) |  |  |
| Intraventricular hemorrhage (IVH) | Yes | 142(61.472) | 56(56.000) | 0.869 | 0.351 |
|  | No | 89(38.528) | 44(44.000) |  |  |
| Concomitant intracerebral hemorrhage (ICH) | No | 204 (88.312) | 86 (86.000) | 0.344 | 0.558 |
|  | Yes | 27(11.688) | 14 (14.000) |  |  |
| Postoperative lumbar puncture | No | 30(12.987) | 14 (14.000) | 0.062 | 0.803 |
|  | Yes | 201 (87.013) | 86 (86.000) |  |  |
| Postoperative lumbar cistern drainage | No | 218(94.372) | 97 (97.000) | 1.048 | 0.306 |
|  | Yes | 13(5.628) | 3(3.000) |  |  |
| Postoperative cerebrospinal fluid GLU concentration, mmol/L |  | 3.020 [2.250, 3.610] | 3.130[2.360,3.695] | -0.354 | 0.724 |
| Postoperative cerebrospinal fluid pro content, mg/L |  | 909.090 [512.720, 1878.780] | 983.680 [549.740, 1805.010] | 0.346 | 0.729 |
| GCS score at admission |  | 14.000[9.000,15.000] | 13.000[7.000,15.000] | 1.707 | 0.081 |

**Table S4. Optimal Cutoff Values for GCS Score at Admission and CSF Biochemical Markers with Bootstrap Validation**

| Feature | Judeon Index (95% CI) | Optimal Threshold | Bootstrap 95% CI (n=1000) |
| --- | --- | --- | --- |
| Admission GCS Score | 0.565 (0.462–0.688) | 11.000 (9.000–13.525) | (9.500–13.000) |
| Postoperative CSF GLU concentration | 0.209 (0.123–0.354) | 2.000 (1.940–3.020) | (1.865–2.355) |
| Postoperative CSF protein (Pro) level | 0.301 (0.235–0.447) | 1226.990 (735.350–1963.347) | (898.500–1650.200) |

**Table S5. LASSO Regression Independent Variable Selection and Coefficients**

| Independent Variables | Minimum Mean Squared Error Coefficient | Standard Error of Minimum Distance Coefficient |
| --- | --- | --- |
| GCS score at admission | -0.357 | -0.386 |
| Hunt-Hess classification at admission | 0.386 | 0.179 |
| Fisher classification at admission | 0.265 | 0.0 |
| Aneurysm size | 0.033 | 0.0 |
| Surgical approach | 0.0 | 0.0 |
| Terminal plate stoma | 0.236 | 0.0 |
| Lateral ventriculostomy | -1.014 | -0.758 |
| Presence of Ventricular Hemorrhage | 1.089 | 1.02 |
| Whether accompanied by parenchymal hemorrhage | 0.0 | 0.0 |
| Postoperative lumbar puncture | 0.0 | 0.0 |
| Postoperative lumbar cistern drainage | 0.74 | 0.0 |
| Postoperative CSF GLU content | -0.175 | 0.0 |
| Postoperative CSF protein (Pro) level | 0.0 | 0.0 |

**Table S6 Comparison of Calibration Performance Metrics Across Models on the locked Test Set**

| Model | Brier Score (95% CI) | Calibration Slope (95% CI) | Calibration Intercept (95% CI) |
| --- | --- | --- | --- |
| Logistic | 0.128 (0.101–0.158) | 0.95 (0.72–1.18) | -0.08 (-0.31-0.15) |
| XGBoost | 0.141 (0.112–0.173) | 0.82 (0.61-1.03) | 0.12 (-0.10-0.34) |
| LightGBM | 0.139 (0.110–0.171) | 0.85 (0.64–1.06) | 0.09 (-0.13 to 0.31) |
| Random Forest | 0.155 (0.125–0.188) | 0.71 (0.52–0.90) | 0.25 (0.07–0.43) |
| AdaBoost | 0.147 (0.118–0.179) | 0.78 (0.58-0.98) | 0.16 (-0.05-0.37) |
| SVM | 0.149 (0.120–0.181) | 0.76 (0.56–0.96) | 0.18 (-0.03-0.39) |
| KNN | 0.152 (0.122–0.184) | 0.73 (0.54–0.92) | 0.21 (0.03–0.39) |
| GNB | 0.163 (0.132–0.197) | 0.65 (0.47–0.83) | 0.30 (0.13–0.47) |

**Table S7. Bootstrap optimism-corrected performance of the final logistic regression model**

| Metric | Apparent performance | Mean optimism | Optimism-corrected performance |
| --- | --- | --- | --- |
| AUC | 0.902 | 0.022 | 0.880 |
| Accuracy | 0.735 | 0.025 | 0.710 |
| Sensitivity | 0.889 | 0.031 | 0.858 |
| Specificity | 0.698 | 0.021 | 0.677 |
| Positive predictive value | 0.394 | 0.018 | 0.376 |
| Negative predictive value | 0.967 | 0.006 | 0.961 |
| F1 score | 0.546 | 0.023 | 0.523 |
| Brier score | 0.132 | 0.009 | 0.141 |

**Table S8. Sensitivity analysis comparing dichotomized and continuous GCS models**

| Model | GCS specification | AUC | Accuracy | Sensitivity | Specificity | PPV | NPV | F1 score | Brier score | Calibration slope | Calibration intercept |
| --- | --- | --- | --- | --- | --- | --- | --- | --- | --- | --- | --- |
| Primary model | Dichotomized at 11 | 0.891 | 0.720 | 0.889 | 0.683 | 0.381 | 0.966 | 0.533 | 0.132 | 0.94 | −0.03 |
| Sensitivity model | Continuous GCS | 0.887 | 0.720 | 0.861 | 0.695 | 0.392 | 0.955 | 0.538 | 0.139 | 0.91 | −0.04 |
